# A multi-center cardiovascular magnetic resonance network for tele-training, tele-supervision and knowledge sharing

**DOI:** 10.1186/1532-429X-17-S1-P228

**Published:** 2015-02-03

**Authors:** Fabian Muehlberg, Florian von Knobelsdorff-Brenkenhoff, Daniel Neumann, Julius Traber, Nils Alwardt, Jeanette Schulz-Menger

**Affiliations:** ECRC, Charité University Medicine Berlin and HELIOS Clinics, Berlin, Germany; Helios IT Services GmbH, Berlin, Germany

## Background

Training of cardiovascular magnetic resonance (CMR) is an important topic in times of growing acceptance of the method for accurate diagnosis and management of cardiovascular disease. However, off-site trainings are becoming less acceptable with increasing cost and time pressure. Here we introduce a novel CMR network, capable of partially remote CMR training and continuous remote expert support.

## Methods

Conceptual, technical and content-related characteristics of our teaching methods are introduced. 97 participants of traditional fellowship CMR teaching and the novel module-based network teaching were surveyed to assess their CMR performance.

## Results

**Figure 1:**
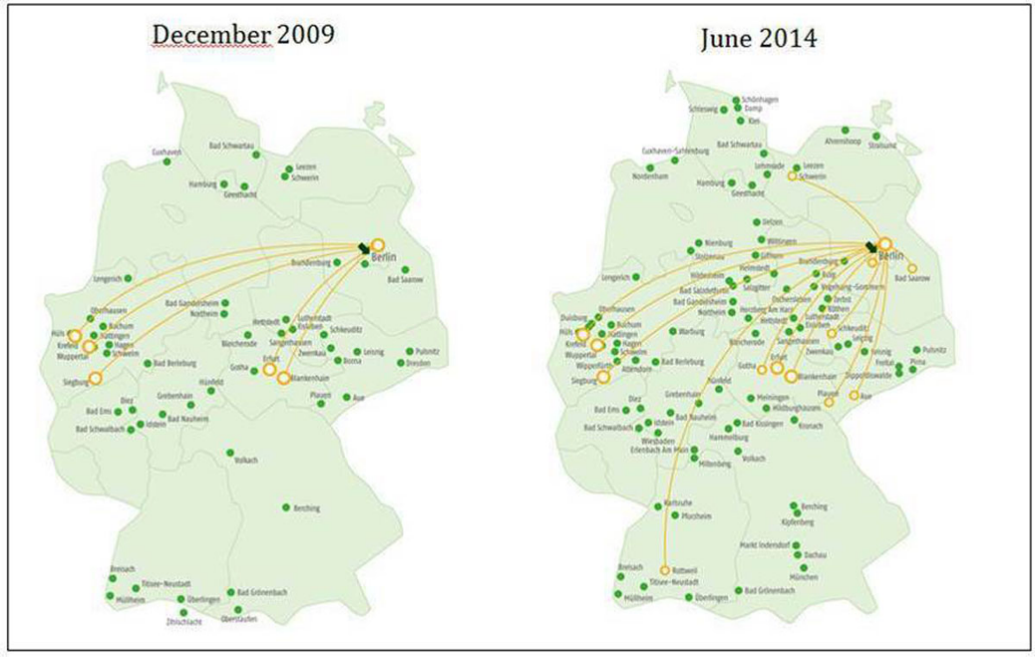
CMR network expansion between December 2009 and June 2014.

**Figure 2:**
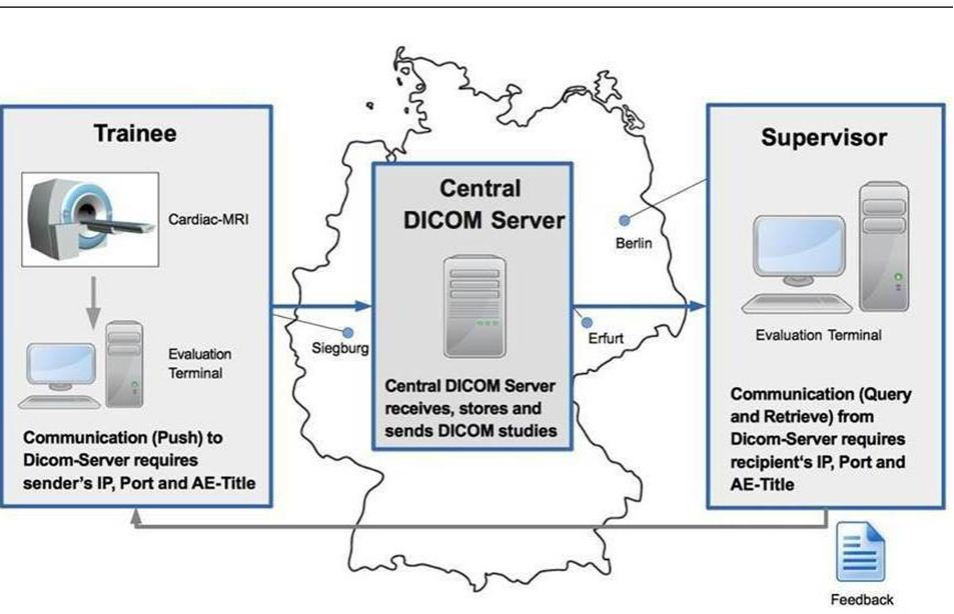
Illustration of the CMR network architecture.

The number of hospitals in our CMR network increased from 5 in 2009 to 14 in 2014. 79% of network hospitals conducted more than 100 CMR scans annually. (Fig. 1) Among these network hospitals are four small institutions (< 400 beds) and five medium-sized hospitals (400-1000 beds). Network teaching reduced off-site CMR training to only five weeks. The time to first self-conducted CMR scans was one week in network teaching but more than one month for 32% of participants in traditional CMR teaching. The CMR network enables experts from distant locations to supervise and control CMR scans in a distant hospital in real-time. (Fig. 2)

## Conclusions

A CMR network can be built with reasonable amount of technical and man power-related resources. It provides an efficient teaching platform with minimum off-site time for trainees. Real-time remote supervision and scan control capabilities support the decentralization of CMR expertise and enable even small and rurally located institutions to offer CMR scans at high-quality level.

